# Unsteady natural convection flow of blood Casson nanofluid (Au) in a cylinder: nano-cryosurgery applications

**DOI:** 10.1038/s41598-023-30129-6

**Published:** 2023-04-09

**Authors:** Wan Faezah Wan Azmi, Ahmad Qushairi Mohamad, Lim Yeou Jiann, Sharidan Shafie

**Affiliations:** Department of Mathematical Sciences, Faculty of Science, Universiti Teknologi Malaysia, 81310 UTM Johor Bahru, Johor Malaysia

**Keywords:** Cancer, Mathematics and computing, Nanoscience and technology

## Abstract

Nano-cryosurgery is one of the effective ways to treat cancerous cells with minimum harm to healthy adjacent cells. Clinical experimental research consumes time and cost. Thus, developing a mathematical simulation model is useful for time and cost-saving, especially in designing the experiment. Investigating the Casson nanofluid's unsteady flow in an artery with the convective effect is the goal of the current investigation. The nanofluid is considered to flow in the blood arteries. Therefore, the slip velocity effect is concerned. Blood is a base fluid with gold (Au) nanoparticles dispersed in the base fluid. The resultant governing equations are solved by utilising the Laplace transform regarding the time and the finite Hankel transform regarding the radial coordinate. The resulting analytical answers for velocity and temperature are then displayed and visually described. It is found that the temperature enhancement occurred by arising nanoparticles volume fraction and time parameter. The blood velocity increases as the slip velocity, time parameter, thermal Grashof number, and nanoparticles volume fraction increase. Whereas the velocity decreases with the Casson parameter. Thus, by adding Au nanoparticles, the tissue thermal conductivity enhanced which has the consequence of freezing the tissue in nano-cryosurgery treatment significantly.

## Introduction

Non-Newtonian fluids, also known as viscous complex fluids, attracted many researchers to study since their rheological properties of physiological being broadly used in the industry and technology applications^1^. It has a nonlinear correlation or consisting of yield stress between shear stress and strain rate because it defies Newton's Law of Viscosity. Many biological fluids exhibit non-Newtonian behaviour, according to earlier investigations^2^. The non-Newtonian fluid widely recognised with a distinctive behaviour is Casson fluid^3–6^. No flow will occur if yield stress is smaller than externally applied shear stress and vice versa. Numerous researchers have utilised it to represent blood flow in small arteries. Venkatesan et al.^7^ highlighted that it is appropriate for representing the basic shear behaviour of blood in a small blood vessel with diameters ranging from 130 to 1000 µm. The fluids are also known as heat carriers in the heat transfer process.

Besides that, natural convection or free convection is one category of heat transfer process which is dependent on fluid motion. It involves the movement of fluid masses, buoyancy, and gravity forces. The heat transfer process occurs if the fluid is driven naturally due to the temperature gradient. It is important in a variety of contexts, such as cooling a nuclear reactor, bioheat models like blood flow inside arteries, thermal storage systems, solar energy systems in solar water heaters, and others^8–10^. Due to its significant applications, many scholars are eager to learn more about natural convection flow in different types of geometrical conditions, either in a transient or unsteady state, such as plate, channel, cylinder, and many more. Examples of the study for natural convection flow on the plate had been done by Khalid et al.^11–13^, which investigated the behaviour of Casson fluid and analytically obtained the solution by using the Laplace transform method. Khan et al.^14^ expanded the issue by adding the shear stress impact. Then, Kataria et al.^15^ gained exact solutions for a similar problem as Khalid et al. with additional MHD and porous medium effects. Abdelhameed^16^ studied the Newtonian fluid on an accelerated plate with the MHD effect. Besides that, the natural convection flow of the various type of fluid between two vertical plates or passed through a channel has attracted researchers to investigate further its behaviour since it represents a cross-sectional cylinder. Narahari and Vijay^17^ and Mohamad et al.^18^ considered the Newtonian fluid and Casson fluid flow past through a fixed channel. To resolve the issues, they all applied the Laplace transform technique. The results revealed that the Casson parameter had a diminishing influence on velocity due to rise in the fluid viscosity. To have better understanding the natural convection flow problems closer to the real-life applications, researchers solved the problems in the cylindrical domain. For example, Shah et al.^19^ resolved the problem analytically for the Newtonian fluid in a fixed cylinder with MHD and pressure gradient effects. Khan et al., Ahmed et al., and Javaid et al.^20–22^ explained the behaviour of the fluid flow in an oscillating cylinder with Newtonian fluid and second-grade fluid. After that, various boundary conditions were used to study the Casson fluid in a cylinder by Ali et al.^23–25^. They all used a combination of the Laplace and Hankel transform approaches to overcome the issues. They discovered that velocity profiles rise when the Casson parameter increases. It is due to yield stress drop and thinning of the boundary layer.

To enhance the effectiveness and efficiency of the heat transfer system, nanotechnology is introduced by suspending metallic and non-metallic nanoparticles in conventional fluids, which are known as nanofluids and were first introduced by Choi^22^. This enhancement method is important in many industries sector, such as biomedical, automotive, aerospace, petrochemical, and manufacturing^26–28^. The term "nanofluid" refers to a fluid adequately disseminated in conventional heat transfer fluids and contains solid nanoparticles or nanofibers with small volumetric amounts of nanometer-sized particles. Water, alcohol, and lubricating oil are examples of common heat transfer fluids with low thermal conductivity. Thus, nanofluid with solid metallic particles is an efficient method for enhancing the heat conductivity of typical fluids^29,30^. Several instances of nanoparticles that have been widely utilized in nanofluids are Titanium oxide (TiO_2_), copper (Cu), aluminium (Al), alumina (Al_2_O_3_), carbon nanotube (CNTs), copper oxide (CuO), gold (Au) and silver (Ag). Nanofluids have achieved many advantages, such as increased heat conduction, unobstructed microchannel cooling, improved thermal conductivity, and mixture stability as a result of the molecular chain behaviour^31–36^. Trisaksri and Wongwises^37^ found that the natural convection flow of nanofluids is lower than the base fluids. The instability of the liquid's density distribution because of the thermal gradient and the dispersion of the particle concentration produced by sedimentation are the causes of the occurrence of natural convection of nanoparticles. However, it can be overcome using appropriate particle concentration and density. Noranuar et al.^38,39^ modelled mathematically the natural convection flow of carbon nanotubes dispersed in the water as a Newtonian fluid model and Casson fluid model with non-coaxial rotation of moving disk. They obtained solutions by utilising Laplace transform. They discovered that fluid velocity and temperature rise with the upsurge of nanoparticle volume fraction. Carbon nanotubes are among the maximum thermal conductivity. Besides that, Saqib et al.^40^ investigated the same nanofluids with a different fluid model, the Brinkman type of fluid model, which flows over an oscillating plate with an MHD effect. They obtained the same result as Noranuar et al.^38^ for the blood temperature but oppositely for the blood velocity due to the heat conductivity and density factors of carbon nanotubes. Sarwar et al.^41^ explored the behaviour of blood flow as the Newtonian fluid with the dispersion of copper nanoparticles in the arteries. They solved the problem numerically and concluded that nanoparticle causes blood flow and temperature increase. Hamarsheh et al.^42^ studied nanoparticles similar to Saqib et al.^40^ with graphite oxide as a base fluid using Casson fluid model to solve the problem in a fixed cylinder numerically. Then, Ahmad et al.^43^ solved analytically natural convection flow on a moving plate and used the same fluid model as Hamarsheh et al. It exhibited that velocity for Casson fluid shows more restraint compared to the viscous fluid. Thus, this technology is important in several industrial fields such as production, energy generation, chemical, metallurgy, electronics and health sciences like cancer treatment by using heat therapy^44^.

Recently, the thermal characteristics of bio-nanofluids like blood with nanoparticle suspension drew interest from scientists since it uses nanotechnology in cancer treatment and medicine, such as hyperthermia method, magnetic resonance imaging (MRI), and nano-cryosurgery^45,46^. Nano-cryosurgery is one of the most effective methods to control and destroy malignant tissue by appropriately freezing with minimum harm to the surrounding healthy tissue, less bleeding, and fewer complications. The tumour tissue needs to be loaded with highly conductive nanoparticles and good biological compatibility to avoid any toxicity. Gold (Au) nanoparticles have the highest freezing efficiency among the nanoparticles that are biologically compatible, like Al_2_O_3_ and Fe_3_O_4_^47–49^. Since it is a lack of clinical experimental research due to time and cost, some researchers developed mathematical modelling to study this matter further. Researchers developed various mathematical modelling for the blood flow with gold nanoparticles, such as Bathi^50^ and Shah et al.^51^ investigated problems in a fixed channel by using the fluid models of Jeffrey and micropolar. Besides that, Khan et al.^52^ and Imtiaz et al.^53^ studied Casson fluid model on stretching radial surface and fixed cylinder.

Most of the researchers solved the problems with different types of boundary geometries and conditions, but none of them assumed the existence of slip effect at the boundary which is a similar assumption of the Navier–Stokes concept. This concept is not applicable to liquids, solutions of polymers, emulsions, and also foams^54^. The slip velocity is a finite velocity between two types of medium. For example, a velocity gradient exists between fluid and boundary. Navier is the earliest researcher who introduced it in 1823, which stated that slip velocity is directly related to boundary shear stress^55,56^. Inspired by him, researchers are interested in studying the slip velocity effects in different geometries and fluids analytically since it exists in real-life applications. Examples study on the effects of slip velocity on the plate are done by Imran et al.^57^, and Saqib et al.^58^ analytically solved the Casson fluid flow on an oscillating plate with the influence of natural convection, radiation, and chemical reaction. Then, Ramesh and Devakar^59^ and Qayyum et al.^60^ discussed the slip velocity effect in a channel of Casson fluid with MHD and porous medium. Later, researchers extend their study in the cylindrical domain with various fluid models since it is close to the actual applications such as Jiang et al.^61^ and Shah et al.^62^ studied Oldroyd-B fluid meanwhile Padma et al.^63,64^ studied Jeffrey fluid model as blood. Besides that, researchers also numerically investigated the slip velocity effect of the nanofluid model in different geometries and fluid models such as nanofluid flow on the plate with Casson nanofluid model^65–68^ and Newtonian nanofluid model^69^, nanofluid flow in a channel with Rabinowitsch nanofluid model^70^, Newtonian carbon nanotubes model^71^ and Casson nanofluid model^72,73^, nanofluid flow in cylindrical domain with Casson nanofluid model^74–76^. None of them solved analytically for Casson nanofluid with slip velocity effect in the cylindrical domain.

As far as the authors know, the analytical solution of blood flow with nanoparticles by using the Casson nanofluid model with slip velocity passed via a horizontal cylinder has not been discussed in the current literature. The thermal study is important since cryosurgery is involved with low-temperature treatment. There are two ways for thermal study, which are clinical and theoretical. Clinical study consumes time and cost but theoretical study produces results for significant physical parameters faster with reasonable cost. Thus, the present work's objective is to determine the influence of slip velocity on the unsteady Casson nanofluid with natural convective in a vertical cylinder by developing mathematical modelling. Casson fluid model replicates blood rheology flow in small arteries due to its unique characteristics. The gold nanoparticle is considered in this study since it is biological compatibility and high thermal conductivity. The hybrid method is used to develop analytical answers for fluid velocity and temperature which are Laplace and finite Hankel transforms. Solutions for important parameters are plotted using Maple with zero-order Bessel functions. Nano-cryosurgery treatment is significant since it produces fewer complications, destroys tumour cells, and protects healthy cells with an appropriate freezing efficiency.

## Problem formulation

**Figure 1:**
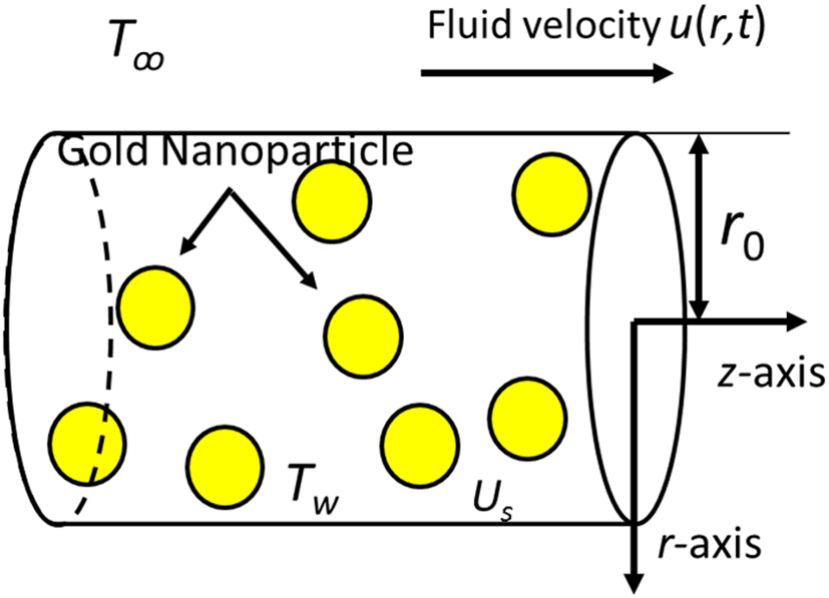
Physical geometry of the fluid flow.

**Figure 2:**
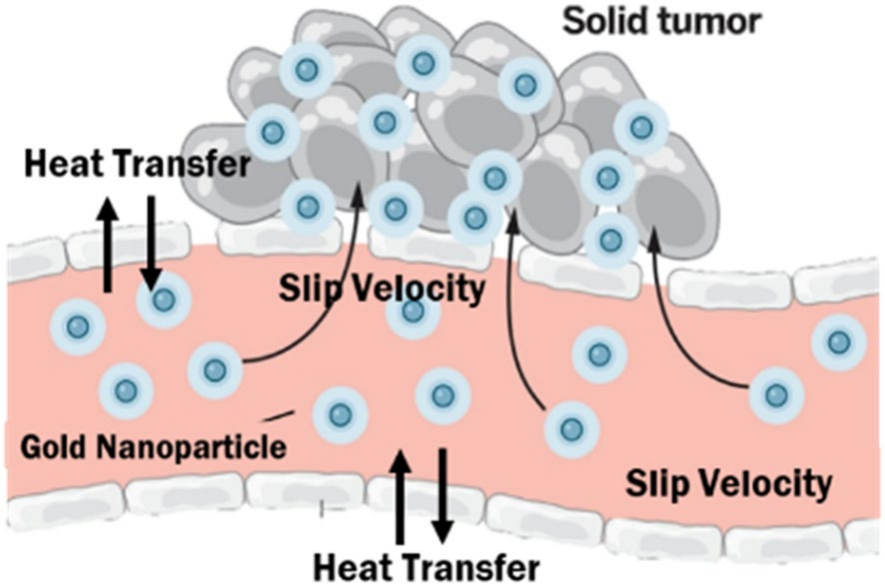
Real application diagram^77^.

The present issue investigates the unsteady and incompressible Casson nanofluid flow in the cylinder. The *z*-axis is considered to run parallel to the cylinder's axis, whereas the *r*-axis is supposed to be perpendicular to it. The radius of the cylinder is considered as *r*_*0*_. Human blood and gold nanoparticles are combined in the Casson nanofluid. The nanofluid flow is driven by buoyancy forces. When t^*^ = 0, both nanofluid, and cylinder are initially at idle condition. The ambient temperature is *T*_ꝏ_. Later, when t^*^ > 0, slip velocity *u*_*s*_ occurs at the cylinder's border, which causes the nanofluid to start flowing. The cylinder temperature was increased simultaneously with the wall cylinder temperature, *T*_*w*_, and then it keeps constant. Assume that *r* and *t* are the sole variables that affect velocity and temperature. Physical details of the issue are shown in Figs. 1 and 2.

The rheological equation of an incompressible Casson fluid which describes the relationship between shear stress and strain rate can be written as follows^9,10,31^

1 $$\tau_{ij} = \left\{ {\begin{array}{*{20}c} {2\left( {\mu_{B} + \frac{{\tau_{y} }}{{\sqrt {2\pi } }}} \right)e_{ij} ;} & {\quad \pi > \pi_{c} } \\ {2\left( {\mu_{B} + \frac{{\tau_{y} }}{{\sqrt {2\pi_{c} } }}} \right)e_{ij} ;} & {\quad \pi < \pi_{c} } \\ \end{array} } \right.$$

where π = *e*_*ij*_*e*_*ij*_ and *e*_*ij*_ are the (*i*,*j*)-th component of the deformation rate, π is the component product of the deformation rate with itself, π_*c*_ is a critical value of this product based on the non-Newtonian model, *µ*_*B*_ is the plastic dynamic viscosity of the non-Newtonian fluid and *τ*_*y*_ is the yield stress of fluid. Under the above assumptions, along with Boussinesq’s approximation and the Tiwari and Das nanofluid model^39^, the governing equations of the momentum and energy for the prescribed study as given by^53^

2 $$\rho_{nf} \frac{{\partial u^{*} }}{{\partial t^{*} }} = \mu_{nf} \left( {1 + \frac{1}{\beta }} \right)\left( {\frac{{\partial^{2} u^{*} }}{{\partial r^{*2} }} + \frac{1}{{r^{*} }}\frac{{\partial u^{*} }}{{\partial r^{*} }}} \right) + g\left( {\rho \beta_{T} } \right)_{nf} \left( {T^{*} - T_{\infty } } \right)$$

and

3 $$\left( {\rho C_{p} } \right)_{nf} \frac{{\partial T^{*} }}{{\partial t^{*} }} = k_{nf} \left( {\frac{{\partial^{2} T^{*} }}{{\partial r^{*2} }} + \frac{1}{{r^{*} }}\frac{{\partial T^{*} }}{{\partial r^{*} }}} \right)$$

as well as the initial and boundary conditions as^21,52^

4 $$\begin{array}{*{20}c} {u^{*} (r^{*} ,\;0) = 0 \, } & {\quad T^{*} \left( {r^{*} ,\;0} \right) = T_{\infty } ;} & {\quad r \in [0,\;r_{0} ],} \\ {\frac{{\partial u^{*} (0,\;t^{*} )}}{{\partial r^{*} }} = 0 \, } & {\quad \frac{{\partial T^{*} (0,\;t^{*} )}}{{\partial r^{*} }} = 0; \, } & { \, \quad t^{*} > 0,} \\ {u^{*} (r_{0}^{*} ,\;t^{*} ) = u_{s}^{*} \, } & {\quad T^{*} \left( {r_{0}^{*} ,\;t^{*} } \right) = T_{w} \, ;} & {\quad \, t^{*} > 0} \\ \end{array}$$

where u^*^ is the velocity component along the *z*-axis, $\beta = {{\mu_{B} \sqrt {2\pi_{c} } } \mathord{\left/ {\vphantom {{\mu_{B} \sqrt {2\pi_{c} } } {\tau_{y} }}} \right. \kern-0pt} {\tau_{y} }}$ is the Casson parameter, *g* is the gravitational acceleration, T^*^ is the temperature of the fluid. Moreover, Eq. (5) are defined the thermophysical properties in Casson nanofluids that have effective density *ρ*_*nf*_, effective heat capacity(*ρc*_*p*_)_*nf*_, effective dynamic viscosity *µ*_*nf*_, effective thermal conductivity *k*_*nf*_ and the thermal expansion coefficient (*ρβ*_*T*_)_*nf*_. According to Oztop et al*.*^78^, the spherical form of nanoparticles is the aspect of the Maxwell model that is taken into account when determining the effectiveness of nanofluids' thermal conductivity. Therefore, using nanofluids is suitable for analysing heat transfer enhancement. Additionally, the base fluid's viscosity which comprises spherical particles is approximately related to the viscosity of the nanofluid had been emphasized by Brickman^48^. Thus, the nanofluid variables are as

5 $$\begin{gathered} \rho_{nf} = \left( {1 - \phi } \right)\rho_{f} + \phi \rho_{s} ,\quad \mu_{nf} = \frac{{\mu_{f} }}{{\left( {1 - \phi } \right)^{2.5} }},\quad k_{nf} = k_{f} \left[ {\frac{{k_{s} + 2k_{f} - 2\phi \left( {k_{f} - k_{s} } \right)}}{{k_{s} + 2k_{f} + \phi \left( {k_{f} - k_{s} } \right)}}} \right], \hfill \\ \left( {\rho \beta_{T} } \right)_{nf} = \left( {1 - \phi } \right)\left( {\rho \beta_{T} } \right)_{f} + \phi \left( {\rho \beta_{T} } \right)_{s} ,\quad \left( {\rho C_{p} } \right)_{nf} = \left( {1 - \phi } \right)\left( {\rho C_{p} } \right)_{f} + \phi \left( {\rho C_{p} } \right)_{s} \hfill \\ \end{gathered}$$

where the subscript symbols *f* and *s* indicate fluid and solid, and *ϕ* is the nanofluid solid volume fraction. The necessary dimensionless variables are shown as^25,64^

6 $$t = \frac{{t^{*} \nu }}{{r_{0}^{2} }},\quad r = \frac{{r^{*} }}{{r_{0} }},\quad u = \frac{{u^{*} }}{{u_{0} }},\quad u_{s} = \frac{{u_{s}^{*} }}{{u_{0} }},\quad \theta = \frac{{T - T_{\infty } }}{{T_{w} - T_{\infty } }}.$$

Employing the dimensionless parameters of Eq. (6) into Eqs. (2–4), now the dimensionless forms of governing equations are obtained as

7 $$\frac{\partial u}{{\partial t}} = a_{1} \beta_{1} \left( {\frac{{\partial^{2} u}}{{\partial r^{2} }} + \frac{1}{r}\frac{\partial u}{{\partial r}}} \right) + a_{2} Gr\theta ,$$

8 $$\frac{\partial \theta }{{\partial t}} = a_{3} \left( {\frac{{\partial^{2} \theta }}{{\partial r^{2} }} + \frac{1}{r}\frac{\partial \theta }{{\partial r}}} \right)$$

with the related dimensionless initial and boundary conditions

9 $$\begin{array}{*{20}c} {u(r,\;0) = 0, \, } & {\quad \theta \left( {r,\;0} \right) = 0;} & {\quad r \in [0,\;1] \, ,} \\ {\frac{\partial u(0,\;t)}{{\partial r}} = 0,} & {\quad \frac{\partial \theta (0,\;t)}{{\partial r}} = 0;} & {\quad t > 0,} \\ {u(1,\;t) = u_{s} ,} & {\quad \theta \left( {1,\;t} \right) = 1;} & {\quad t > 0} \\ \end{array}$$

where $a_{1} = \frac{1}{{\left( {1 - \phi } \right)^{2.5} \left[ {\left( {1 - \phi } \right) + {{\phi \rho_{s} } \mathord{\left/ {\vphantom {{\phi \rho_{s} } {\rho_{f} }}} \right. \kern-0pt} {\rho_{f} }}} \right]}},$ $a_{2} = \frac{{\left( {1 - \phi } \right)\rho_{f} + {{\phi \left( {\rho \beta_{T} } \right)_{s} } \mathord{\left/ {\vphantom {{\phi \left( {\rho \beta_{T} } \right)_{s} } {\left( {\beta_{T} } \right)_{f} }}} \right. \kern-0pt} {\left( {\beta_{T} } \right)_{f} }}}}{{\left( {1 - \phi } \right)\rho_{f} + \phi \rho_{s} }},$ $a_{3} = \frac{{a_{4} }}{{a_{5} \Pr }},$ $a_{4} = \frac{{k_{s} + 2k_{f} - 2\phi \left( {k_{f} - k_{s} } \right)}}{{k_{s} + 2k_{f} + 2\phi \left( {k_{f} - k_{s} } \right)}},$ $a_{5} = \left( {1 - \phi } \right) + \frac{{\phi \left( {\rho c_{p} } \right)_{s} }}{{\left( {\rho c_{p} } \right)_{f} }},$ $\beta_{1} = \frac{1}{{\beta_{0} }} \,$ and $\beta_{0} = 1 + \frac{1}{\beta }$ are the constant parameters. The dimensionless parameters such as Prandtl number and Grashof number are defined as ${\text{Pr}} = \frac{{\nu_{f} \left( {\rho c_{p} } \right)_{f} }}{{k_{f} }}$ and $Gr = \frac{{g\left( {\beta_{T} } \right)_{f} \left( {T_{w} - T_{\infty } } \right)r_{0}^{2} }}{{\nu_{f} u_{0} }}$.

## Problem solution

**Figure 3:**
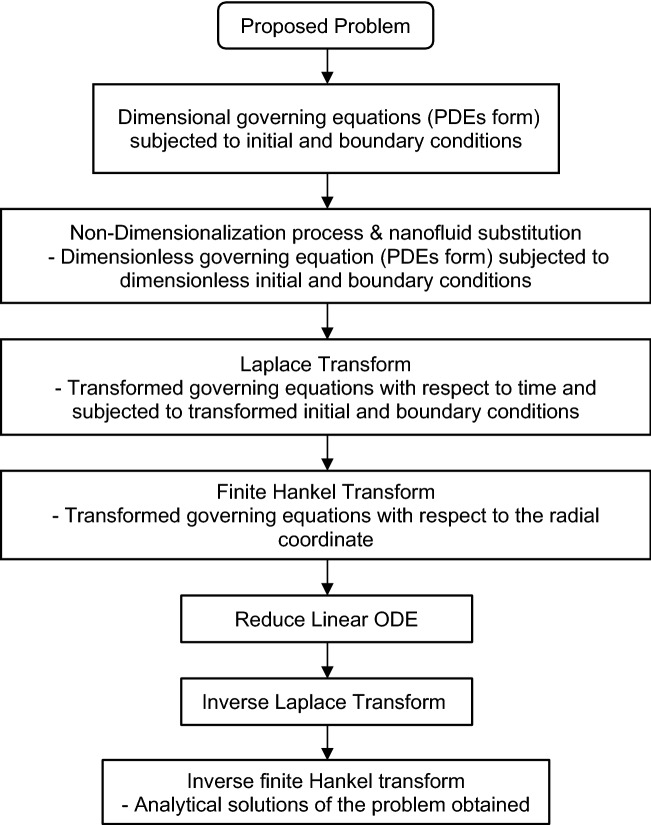
Methodology flowchart.

The problem is modeled with the dimensional partial differential equation (PDE) of the momentum and energy together with the initial and boundary conditions. The nanofluid variables based on the Tiwari and Das models are proposed. The dimensional governing equations with initial and boundary conditions are converted into dimensionless forms by employing dimensionless variables. To reduce the dimensionless PDE to ODE, the Laplace transform is applied with respect to time and the finite Hankel transform with respect to the radial coordinate. Later, the inverse Laplace transform and inverse finite Hankel transform are used to attain the analytical solutions of the velocity and temperature. Both transformation tools are important since the Laplace transform solves initial boundary and transient problems while the finite Hankel transform solves the cylindrical domain problem. Figure 3 displays the flow of the methodology to simplify the method of the problem.

### Solution of the energy equation

Employing the Laplace transform into energy equation (8) and boundary condition (9) subjected to the initial condition (9), yields

11 $$s\overline{\theta } (r,\;s) = a_{3} \left[ {\frac{{\partial^{2} \overline{\theta } (r,\;s)}}{{\partial r^{2} }} + \frac{1}{r}\frac{{\partial \overline{\theta } (r,\;s)}}{\partial r}} \right],$$

12 $$\overline{\theta } (1,\;s) = \frac{1}{s}$$

where $\overline{\theta } (r,\;s)$ is the Laplace transform of the function $\theta (r,\;t)$ and *s* is the transformation variable. Then, applying finite Hankel transform of zero-order to Eq. (11) and by using condition (12), we have

13 $$\overline{{\theta_{H} }} (r_{n} ,\;s) = \left( {\frac{{a_{3} r_{n}^{2} }}{{s\left( {s + a_{3} r_{n}^{2} } \right)}}} \right)\frac{{J_{1} \left( {r_{n} } \right)}}{{r_{n} }}$$

where $\overline{\theta }_{H} (r_{n} ,\;s) = \int_{0}^{1} {r\overline{\theta } (r,\;s)J_{0} \left( {rr_{n} } \right)dr}$ is the finite Hankel transform of the function $\overline{\theta } (r,\;s)$ and *r*_*n*_ with *n* = 0,1,… are the positive roots of the equation $J_{0} (x) = 0$, where *J*_*0*_ is being the Bessel function of the first kind, and zero-order and *J*_*1*_ is the Bessel function of the first kind and first order. By applying partial fraction decomposition on the right-hand side of the Eq. (13), yields

14 $$\overline{{\theta_{H} }} (r_{n} ,\;s) = \left( {\frac{1}{s} - \frac{1}{{s + a_{3} r_{n}^{2} }}} \right)\frac{{J_{1} \left( {r_{n} } \right)}}{{r_{n} }}.$$

Next, the inverse Laplace transform of Eq. (14), gives

15 $$\theta_{H} (r_{n} ,\;t) = \frac{{J_{1} \left( {r_{n} } \right)}}{{r_{n} }}\left( {1 - \exp \left( { - a_{3} r_{n}^{2} t} \right)} \right).$$

Finally, the inverse finite Hankel transform of order zero is given by^79^

16 $$H_{0}^{ - 1} \theta_{H} \left( {r_{n} ,\;t} \right) = \theta \left( {r,\;t} \right) = 2\sum\limits_{n = 1}^{\infty } {\frac{{J_{0} \left( {rr_{n} } \right)}}{{J_{1}^{2} \left( {r_{n} } \right)}}} \theta_{H} \left( {r_{n} ,\;t} \right),$$

is applied to the Eq. (15), and the analytical solution of the temperature profile is written as

17 $$\theta (r,\;t) = 1 - 2\sum\limits_{n = 1}^{\infty } {\frac{{J_{0} \left( {rr_{n} } \right)}}{{r_{n} J_{1} \left( {r_{n} } \right)}}} \exp \left( { - a_{3} r_{n}^{2} t} \right).$$

### Solution of the velocity equation

The Laplace transform is applied to the momentum equation (7) and boundary condition (9), which yields

18 $$s\overline{u}(r,\;s) = a_{1} \beta_{1} \left[ {\frac{{\partial^{2} \overline{u}(r,\;s)}}{{\partial r^{2} }} + \frac{1}{r}\frac{{\partial \overline{u}(r,\;s)}}{\partial r}} \right] + a_{2} Gr\overline{\theta } \left( {r,\;s} \right),$$

19 $$\overline{u}(1,\;s) = \frac{{u_{s} }}{s}$$

where $\overline{u} (r,\;s)$ is the Laplace transform of the function $u(r,t)$. Then, employing finite Hankel transform of zero-order to the Eq. (18) and by using boundary condition (19), give

20 $$\overline{u}_{H} (r_{n} ,\;s) = \left[ {a_{1} \beta_{1} r_{n}^{2} \frac{{u_{s} }}{s} + a_{2} Gr\left( {\frac{1}{s} - \frac{1}{{s + a_{3} r_{n}^{2} }}} \right)} \right]\frac{{J_{1} \left( {r_{n} } \right)}}{{r_{n} }}\frac{1}{{s + a_{1} \beta_{1} r_{n}^{2} }}$$

where $\overline{u}_{H} (r_{n} ,\;s) = \int_{0}^{1} {r\overline{u}(r,\;s)J_{0} \left( {rr_{n} } \right)dr}$ is the finite Hankel transform of the function $\overline{u}(r,\;s)$. Then, to simplify the Eq. (20), partial fraction decomposition is applied to the right-hand side of the equation and obtains

21 $$\overline{u}_{H} \left( {r_{n} ,\;s} \right) = \left[ {\overline{u}_{H1} \left( {r_{n} ,\;s} \right) + a_{2} Gr\left( {\overline{u}_{H2} \left( {r_{n} ,\;s} \right) + \overline{u}_{H3} \left( {r_{n} ,\;s} \right)} \right)} \right]\frac{{J_{1} \left( {r_{n} } \right)}}{{r_{n} }}$$

where $$\overline{u}_{H1} \left( {r_{n} ,\;s} \right) = \frac{{u_{s} }}{s} - \frac{{u_{s} }}{{s + a_{1} \beta_{1} r_{n}^{2} }},$$ $$\overline{u}_{H2} \left( {r_{n} ,\;s} \right) = \frac{1}{{a_{1} \beta_{1} r_{n}^{2} }}\left( {\frac{1}{s} - \frac{1}{{s + a_{1} \beta_{1} r_{n}^{2} }}} \right),$$ $$\overline{u}_{H3} \left( {r_{n} ,\;s} \right) = \frac{1}{{r_{n}^{2} \left( {a_{1} \beta_{1} - a_{3} } \right)}}\left( {\frac{1}{{s + r_{n}^{2} a_{3} }} - \frac{1}{{s + a_{1} \beta_{1} r_{n}^{2} }}} \right).$$

The inverse Laplace transform of the Eq. (21) is

22 $$u_{H} \left( {r_{n} ,\;t} \right) = \frac{{J_{1} \left( {r_{n} } \right)}}{{r_{n} }}\left[ {u_{H1} \left( {r_{n} ,\;t} \right) + a_{2} Gr\left( {u_{H2} \left( {r_{n} ,\;t} \right) - u_{H3} \left( {r_{n} ,\;t} \right)} \right)} \right]$$

with

23 $$u_{H1} \left( {r_{n} ,\;t} \right) = u_{s} \left( {1 - \exp \left( { - a_{6} t} \right)} \right),$$

24 $$u_{H2} \left( {r_{n} ,\;t} \right) = \frac{1}{{a_{6} }}\left( {1 - \exp \left( { - a_{6} t} \right)} \right),$$

25 $$u_{H3} \left( {r_{n} ,\;t} \right) = \frac{1}{{a_{6} - a_{7} }}\left( {\exp \left( { - a_{7} t} \right) - \exp \left( { - a_{6} t} \right)} \right),$$

where $a_{6} = a_{1} \beta_{1} r_{n}^{2} \,$ and $a_{7} = a_{3} r_{n}^{2}$. The inverse finite Hankel transform or zero order is given as

26 $$H_{0}^{ - 1} u_{H} \left( {r_{n} ,\;t} \right) = u\left( {r,\;t} \right) = 2\sum\limits_{n = 1}^{\infty } {\frac{{J_{0} \left( {rr_{n} } \right)}}{{J_{1}^{2} \left( {r_{n} } \right)}}} u_{H} \left( {r_{n} ,\;t} \right),$$

is employing to Eq. (22), and the analytical solution of the nanofluid velocity is obtained as

27 $$u\left( {r,\;t} \right) = u_{s} + 2\sum\limits_{n = 1}^{\infty } {\frac{{J_{0} \left( {rr_{n} } \right)}}{{r_{n} J_{1} \left( {r_{n} } \right)}}} \left[ { - u_{1} \left( {r,\;t} \right) + u_{2} \left( {r,\;t} \right)} \right]$$

with

28 $$u_{1} \left( {r,\;t} \right) = u_{s} \exp \left( { - a_{6} t} \right),$$

29 $$u_{2} \left( {r,\;t} \right) = a_{2} Gr\left( {\frac{1}{{a_{6} }} - \frac{{\exp \left( { - a_{7} t} \right)}}{{a_{6} - a_{7} }} + \frac{{a_{3} \exp \left( { - a_{6} t} \right)}}{{a_{6} \left( {a_{1} \beta_{1} - a_{3} } \right)}}} \right).$$

## Discussion

In this study, the heat transfer issue on the Casson fluid as human blood with the dispersion of the gold nanoparticles in a cylinder. The slip velocity effect is considered to mimic as close as the physical applications. It was solved by utilizing the hybrid technique of the Laplace transform, and finite Hankel transform. The parameters which reflect the change in velocity and temperature have been studied numerically by plotting graphs in Maple software. The influences of several constraints such as Casson parameter *β*, Grashof number *Gr*, nanoparticles volume fraction *ϕ*, slip velocity *u*_*s,*_ and time parameter *t* are analysed in Figs. 5, 6, 7, 8, 9, 10, 11 and 12. The plotting is displayed for two different values of time *t* = 0.5 and *t* = 2.5 to compare and analyse the fluid flow behaviour at the initial state and steady state. Besides, *r* = 0 indicates the centre of the cylinder, while *r* = 1 indicates the wall of the cylinder.

**Figure 4:**
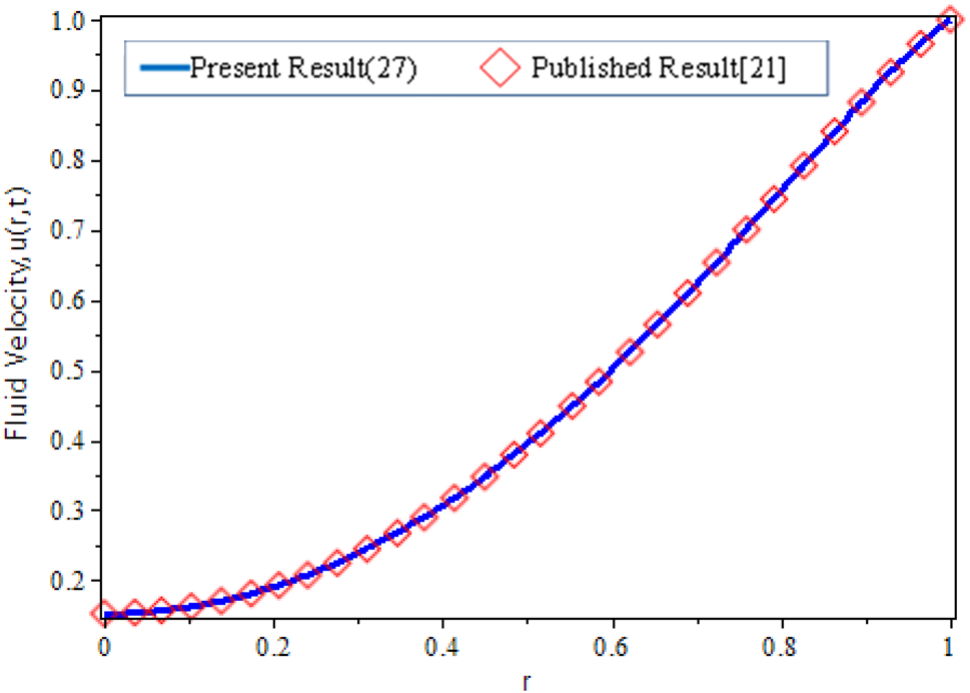
Validation current work with the published result^21^.

To validate the accuracy of the present result in Eq. (27), it will compare with the earlier study done by Khan et al.^21^ as exposed in Fig. 4. The identical velocity profiles are obtained by allowing the parameter of nanoparticle volume fraction *ϕ* = 0, Casson parameter *β* → ꝏ and *u*_*s*_ = 1 in the current result, and by allowing *ω* = 0 in the published results.

Table 1 shows the thermophysical characteristics of blood as a base fluid and gold as nanoparticles. In numerical computations, the physical parameters have been set as^18,48,68^: *β* = 0.8, *ϕ* = 0.2, *Gr* = 1.0, *ν*_*f*_ = 3 × 10^–3^, and the varied parameter values are shown in the figures.

**Table 1:** Thermophysical parameters of blood and gold nanoparticles^39,53^.

Material	Symbol	*ρ *(kg/m^3^)	*C*_*p*_ (J/kg K)	*k* (W/mK)	*β*_*T*_ × 10^–5^ (1/K)
Blood	–	1063	3594	0.492	0.18
Gold	Au	19,300	129	318	1.4

### Blood with nanoparticles flows at the cylinder’s wall

**Figure 5:**
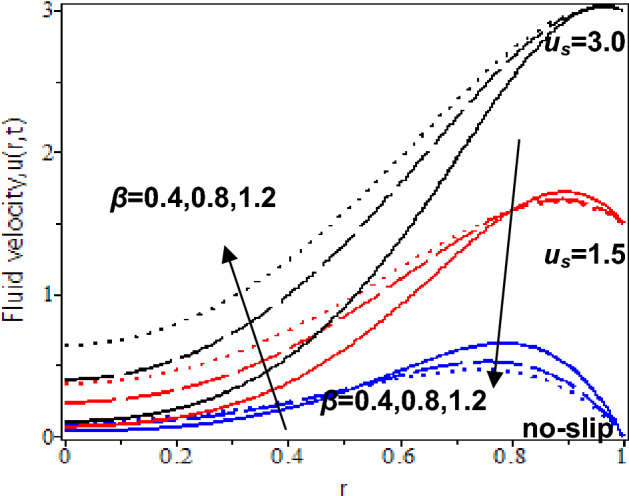
Nanofluid velocity with various Casson parameter and slip velocity at *t* = 0.5.

**Figure 6:**
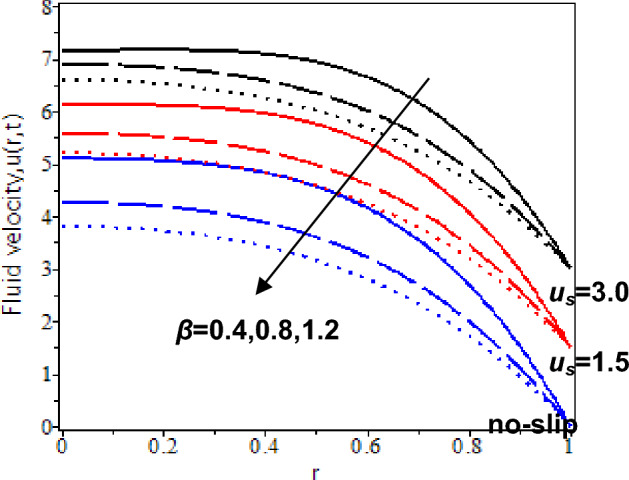
Nanofluid velocity with various Casson parameter and slip velocity at *t* = 2.5.

Figures 5 and 6 show the influence of the Casson parameter on the velocity profiles at the cylinder wall when the slip and no-slip velocity effects are present. It showed that increase in slip velocity would enhance the blood velocity with nanoparticles at the cylinder’s wall. At *t* = 0.5, as the Casson parameter decreases, blood velocity increases for no-slip and small slip velocity effects while blood velocity decreases for larger slip velocity effects. At *t* = 2.5, blood velocity increases when Casson parameters decrease with the slip and no-slip effects. It is because the shear thickening factor reduces as the Casson parameter decreases. It results in thinning the blood flow and enhances blood velocity.

**Figure 7:**
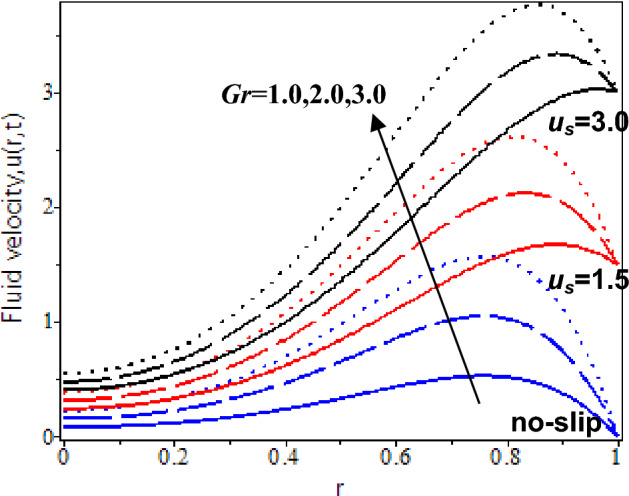
Nanofluid velocity with various Grashof number and slip velocity at *t* = 0.5

**Figure 8:**
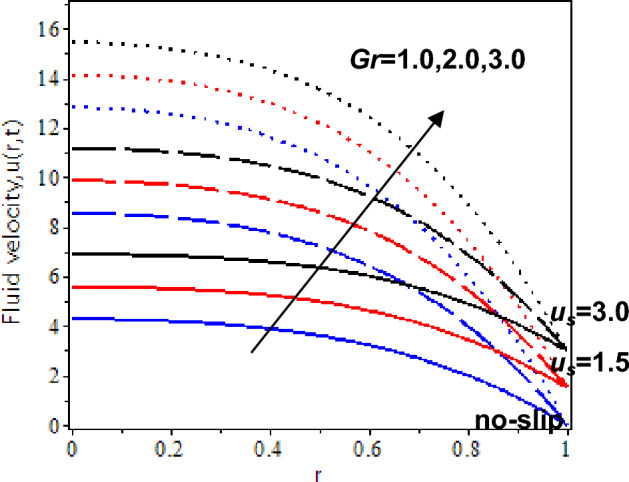
Nanofluid velocity with various Grashof number and slip velocity at *t* = 2.5

Besides that, Figs. 7 and 8 illustrate the nanofluid flow with the effects of thermal Grashof number at the cylinder’s wall with slip and no-slip at the boundary. Based on the observations at *t* = 0.5, blood flows faster when the Grashof number rises for the existence of no-slip and small slip velocity. It is because of the existence of a small thermal buoyancy force and a larger slip velocity effect which is more dominant in influencing the blood velocity at the wall. When* t* = 2.5, the increment of the Grashof number and slip velocity effect led to the increment of blood velocity with nanoparticles.

**Figure 9:**
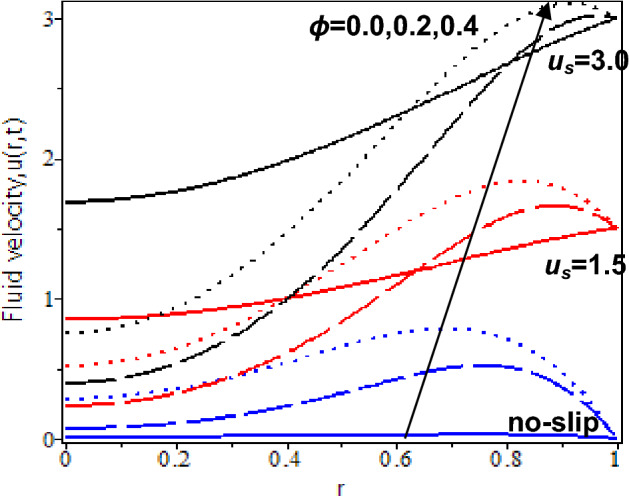
Nanofluid velocity with various nanoparticles volume fraction and slip velocity at *t* = 0.5.

**Figure 10:**
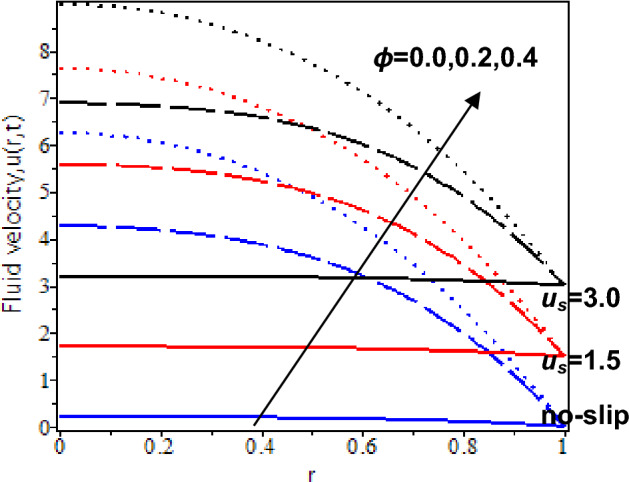
Nanofluid velocity with various nanoparticles volume fraction and slip velocity at *t* = 2.5.

Furthermore, the influences of nanoparticles volume fraction in the blood flow with the presence of slip and no-slip at the cylinder’s wall are shown in Figs. 9 and 10. At *t* = 0.5, it is reflected that when *ϕ* = 0 (pure blood flow without nanoparticles), blood velocity is slightly increasing with the no-slip velocity effect while it decreases with the existence of the slip velocity effect. Besides, nanoparticles volume fraction increases will cause blood velocity increases for no-slip and slip velocity effects. At *t* = 2.5, the blood velocity without nanoparticles, *ϕ* = 0 is slightly increased for no-slip and slip conditions. However, an increment of the nanoparticles volume fraction and slip velocity effect will cause an increment in the blood velocity due to a rise in the energy transmission by blood flow. In other words, increasing the nanoparticles' volume fraction will enhance the thermal dispersion in the nanofluid flow and lead to the improvement of blood velocity. It is supported by the previous study that nanoparticles greatly accelerated with time and contributed to the ice ball formation, which is a vital aspect in killing the targeted tumor. Hence, its proven nanoparticles enhance nano-cryosurgery treatment^80^.

**Figure 11:**
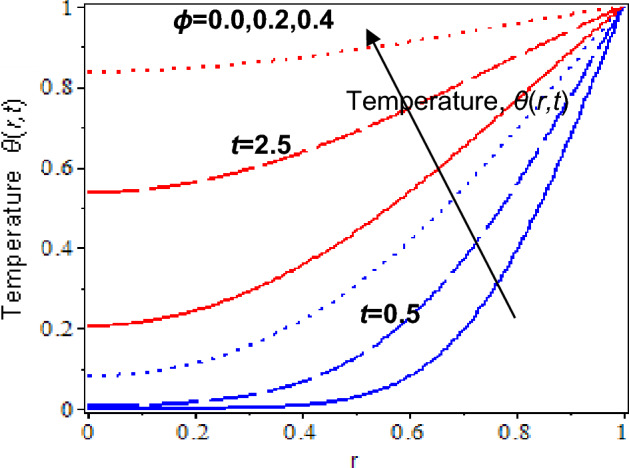
Nanofluid temperature with various nanoparticles volume fraction and time parameter.

**Figure 12:**
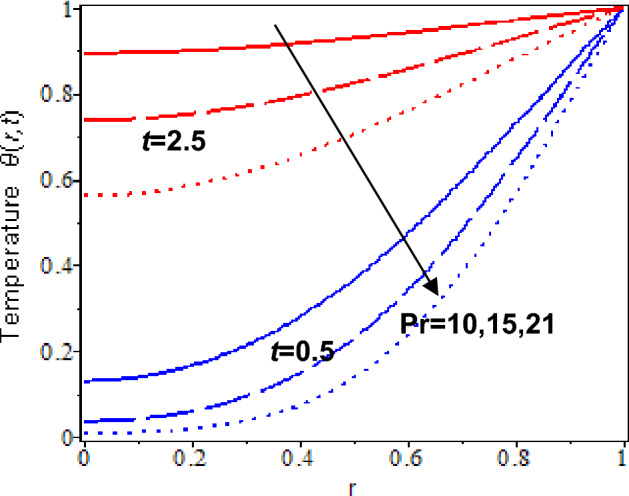
Nanofluid temperature with various Prandtl number and time parameter.

Lastly, the outcome of nanoparticles volume fraction on the temperature profiles at *t* = 0.5 and *t* = 2.5 are exhibited in Fig. 11. Based on the observations, increases in the nanoparticles concentration will result in to increase in blood temperature. It is because gold nanoparticles have a high heat conductivity and cause an increment of the heat transfer rate in the blood flow. Besides, the temperature of blood with nanoparticles decreases as the Prandtl number increases for both times, *t* = 0.5, *t* = 2.5, which displays in Fig. 12. It is because thermal diffusion rises for a larger Prandtl number. It is agreed with the experimental phenomena that it boosts the freezing rate and enlarged ice formation at the targeted tumor in the nano-cryosurgery process^81^.

Thus, in nano-cryosurgery, both obtained velocity and temperature results are significant to enhance the freezing process, optimising the killing of the tumour cell without harming the surrounding healthy tissues. Hence, it can be concluded that blood velocity with nanoparticles at the wall of the cylinder is also influenced by the occurrence of slip velocity effect besides the effects of the Casson parameter, Grashof number, and nanoparticles volume fraction.

### Blood with nanoparticles flows at the cylinder’s centre

The consequences of the Casson parameter, *β* on the blood nanofluid flow as approaching the centre of the cylinder with slip and no-slip velocity are illustrated in Figs. 5 and 6. Based on the observations, when* t* = 0.5, blood velocity with nanoparticles rises as the Casson parameter increases. It is because yield stress of fluid fall. It means that the force needed to start the fluid flow reduces. However, the relation between the Casson parameter and blood velocity is inversely proportional at *t* = 2.5. As we can see, blood velocity with nanoparticles decline as the Casson parameter rises. The increase in Casson parameters will elevate fluid viscosity and produce resistance in the blood flow.

Moreover, Figs. 7 and 8 display the velocity profiles with the result of the thermal Grashof number as approaching the centre of the cylinder with the slip and no-slip velocity. At *t* = 0.5, blood velocity gradually decreases as it approaches the centre of the cylinder. It is due to the viscosity force of the blood being larger at the initial state and when approaching the centre of the cylinder. Besides, blood velocity enhances as the Grashof number rises. When *t* = 2.5, blood velocity increases as the Grashof number is increased. It is because blood density decreases as the Grashof number increases and has less effect on the viscous force, which causes enhancement of the blood velocity.

Additionally, the influences of nanoparticles volume fraction in the blood flow approaching the centre of the cylinder with the slip and no-slip velocity are shown in Figs. 9 and 10. At *t* = 0.5, blood velocity without nanoparticles, *ϕ* = 0 constantly flows towards the centre of the cylinder for the no-slip velocity effect while blood velocity is decreased for the existence of the slip velocity effect. However, pure blood velocity is higher than blood velocity with nanoparticles for the slip velocity effect. It is due to an increase in internal friction force with the blood concentration and slips effect at the boundary. Thus, as nanoparticles volume fraction increases, blood velocity drastically decreases for the larger slip velocity effects. At *t* = 2.5, blood velocity without nanoparticles, *ϕ* = 0 is slightly increased and constantly flows towards the centre of the cylinder. However, blood velocity with nanoparticles rapidly increases as the nanoparticles volume fraction increases. The thermal transfer rate increases when nanoparticle concentration is increased in the blood flow, which leads to blood velocity enhancement. Thus, cryosurgery with the adoption of nanoparticles increases the delivery of anti-cancer drugs during cryosurgery treatment. It also can eliminate cancer tumour cells accurately.

Finally, the impact of nanoparticles volume fraction on the temperature profiles when approaching the centre of the cylinder at *t* = 0.5 and *t* = 2.5 are disclosed in Fig. 11. Blood temperature decreases as it approaches the centre of the cylinder. It is due to the heat transfer rate not being equally distributed from the wall to the centre of the cylinder. However, blood temperature increases as nanoparticle volume fraction and time parameter increase. Figure 12 depicts the influence of the increment Prandtl number on the temperature profile at the cylinder’s centre. At *t* = 0.5, it is clearly shown that temperature profiles decrease as the approach towards the centre for larger Prandtl number, but the temperature profiles slightly decrease at *t* = 2.5. It is because blood temperature cools down quickly for a larger Prandtl number and the heat transfer process reaches an equilibrium state as time larger. Human blood has a larger Prandtl number, and adding nanoparticles helps to increase thermal diffusion in the blood, which is beneficial in ice formation during nano-cryosurgery procedure^81^.

From the obtained results of human blood velocity and temperature above, it is proven that injection of nanoparticles increases the freezing rate in nano-cryosurgery and eliminates the ‘dead zone’ between two cryoprobes due to insufficient freezing^82^. A summarization of the comparison of the results between blood flow at the cylinder’s wall and blood flow at the cylinder’s centre is presented in Table 2.

**Table 2:** Results comparisons for velocity and temperature profiles.

Parameters (increase)	Velocity profiles				Temperature profiles	
	Blood flow at the cylinder’s centre		Blood flow at the cylinder’s wall		Blood flow at the cylinder’s centre	Blood flow at the cylinder’s wall
	t = 0.5	t = 2.5	t = 0.5	t = 2.5		
Casson parameter	Increase for no-slip/slip effects	Decrease for no-slip/slip effects	Decrease (no-slip/small-slip effects) Increase (larger slip effects)	Decrease for no-slip/slip effects		
Thermal Grashof number	Increase for no-slip/slip effects	Increase for no-slip/slip effects	Increase (no-slip / small-slip effects) Decrease (larger slip effect + small Grashof number)	Increase for no-slip/slip effects		
Nanoparticles volume fraction	Decrease for slip effects Increase for no slip effect	Increase for no-slip/slip effects	Increase for no-slip/slip effects	Increase for no-slip/slip effects	Increase	Increase
Prandtl number					Decrease	Decrease
Time					Increase	Increase

## Conclusion

The analytical solutions for the unsteady flow of blood Casson nanofluid with gold nanoparticles in a cylinder with natural convective and slip velocity effects have been investigated. The expressions for fluid velocity and fluid temperature were attained by using the hybrid method of Laplace transform and finite Hankel transform. Present results have been validated with the published results by Khan et al.^21^, and it is found that both comparison results are in mutual agreement. The important discoveries of this study are summarised as follows:

- Blood velocity with nanoparticles at the wall of the cylinder increases with increases of *Gr*, *u*_*s*_, and *ϕ.*
- Increasing values of *β* at the wall of the cylinder causes blood velocity to decrease for no-slip and small-slip velocity effects for the small-time interval.
- As *β* increases at the cylinder’s centre, blood velocity rises for the small-time interval while blood velocity decreases for the larger-time interval.
- An increase of *Gr*,* ϕ* and* t* at the centre of the cylinder led to an increase in velocity profiles.
- Blood velocity with nanoparticles decreases as it approaches the cylinder’s centre for a small-time interval.
- The blood temperature upsurges with the arising values of *ϕ* and *t* while declining with an increment of Pr values.

The significant findings obtained in this study can enhance the freezing rate to help in ice formation and kill the tumor. Moreover, the present study shows that blood velocity enhances with nanoparticles which is important in anti-cancer drug delivery. All the findings benefit nano-cryosurgery applications to treat the cancerous cell with fewer complications effectively. For future research, it is suggested to study the other boundary and effects such as inclined cylinder, radiation heat transfer process, pressure gradient, porous medium, chemical reaction, and other types of nanofluid models.

## Data Availability

All data generated or analysed during this study are included in this published article.
